# Data and sample sharing as an enabler for large-scale biomarker research and development: The EPND perspective

**DOI:** 10.3389/fneur.2022.1031091

**Published:** 2022-11-30

**Authors:** Niranjan Bose, Anthony J. Brookes, Phil Scordis, Pieter Jelle Visser

**Affiliations:** ^1^Health and Life Sciences, Gates Ventures, Kirkland, WA, United States; ^2^Department of Health Metrics Sciences, University of Washington, Seattle, WA, United States; ^3^Department of Genetics and Genome Biology, University of Leicester, Leicester, United Kingdom; ^4^UCB Biopharma UK, Slough, United Kingdom; ^5^Alzheimer Center Amsterdam, Department of Neurology, Amsterdam Neuroscience, Vrije Universiteit Amsterdam, Amsterdam UMC, Amsterdam, Netherlands; ^6^Alzheimer Center Limburg, School for Mental Health and Neuroscience, Maastricht University, Maastricht, Netherlands; ^7^Department of Neurobiology, Care Sciences and Society, Division of Neurogeriatrics, Karolinska Institutet, Stockholm, Sweden

**Keywords:** neurodegenerative disease, Alzheimer's disease, data-sharing, sample-sharing, platforms, biomarker research, cohort

## Abstract

Biomarker discovery, development, and validation are reliant on large-scale analyses of high-quality samples and data. Currently, significant quantities of data and samples have been generated by European studies on Alzheimer's disease (AD) and other neurodegenerative diseases (NDD), representing a valuable resource for developing biomarkers to support early detection of disease, treatment monitoring, and patient stratification. However, discovery of, access to, and sharing of data and samples from AD and NDD research are hindered both by silos that limit collaboration, and by the array of complex requirements for secure, legal, and ethical sharing. In this Perspective article, we examine key challenges currently hampering large-scale biomarker research, and outline how the European Platform for Neurodegenerative Diseases (EPND) plans to address them. The first such challenge is a fragmented landscape filled with technical barriers that make it difficult to discover and access high-quality samples and data in one location. A second challenge is related to the complex array of legal and ethical requirements that must be navigated by researchers when sharing data and samples, to ensure compliance with data protection regulations and research ethics. Another challenge is the lack of broad-scale collaboration and opportunities to facilitate partnerships between data and sample contributors and researchers, in addition to a lack of regulatory engagement early in the research process to enable validation of potential biomarkers. A further challenge facing projects is the need to remain sustainable beyond initial funding periods, ensuring data and samples are shared and reused, thereby driving further research and innovation. In addressing these challenges, EPND will enable an environment of faster and more disruptive research on diagnostics and disease-modifying therapies for Alzheimer's disease and other neurodegenerative diseases.

## Introduction to EPND

Nearly 55 million people around the world live with dementia, with Alzheimer's dementia making up 60 to 70 percent of global cases (1). The second most prevalent neurodegenerative disorder, Parkinson's disease (PD), has impacted more than 8.5 million people globally, and disability and death due to PD is “increasing faster than for any other neurological disorder.” (2). These numbers are only expected to worsen over time, with the Institute for Health Metrics and Evaluation (IHME) forecasting the number of people with dementia will almost triple in the next decades, from 57 million in 2019 to over 152 million cases by 2050 (3). Although they represent the leading causes of disability and dependency globally (1, 2), there exists a high unmet need for effective diagnostics and disease-modifying therapies for neurogenerative diseases. The progression and severity of these diseases vary widely between patients, due in part to the complex underlying pathophysiological mechanisms. Improved and validated diagnostic tests using imaging or fluid-based biomarkers, such as positron emission tomography (PET), cerebrospinal fluid (CSF) and blood tests to detect proteins such as beta-amyloid, tau and alpha-synuclein, could help support early detection of disease, assessment of treatment efficacy, and more accurately stratify patients (4). However, great challenges hinder progress: biomarker discovery, development, and regulatory validation are reliant on large-scale analyses of high-quality data and samples, and currently, discovery of, access to, and sharing of these valuable resources is hindered by varied information ‘silos' that limit collaboration. In addition, there exists an array of complex requirements for secure, legal, and ethical sharing that has impeded much-needed progress in AD and other neurodegenerative disease (NDD) research.

The European Platform for Neurodegenerative Diseases (EPND) project aims to address some of these specific barriers and deliver a scalable and sustainable platform for sample and data sharing that will integrate existing data and sample discovery tools. The project, a public-private partnership that started in late 2021, is a collaboration between the Innovative Medicines Initiative (IMI) and the European Federation of Pharmaceutical Industries and Associations (EFPIA). EPND involves 29 organizations across Europe, the United States, and Israel that are united under a common goal: to change the way NDD research is utilized and accessed to accelerate impact.

EPND will be a platform for data and sample discovery, access, and analysis. It will gather a global community of scientists to advance research for the identification and regulatory validation of biomarkers, and in doing so, facilitate the accelerated development of diagnostics and treatment of AD and other NDDs.

## EPND: Opportunities to accelerate neurodegenerative disease biomarker and therapeutic research

### Harmonizing a fragmented landscape and addressing technical barriers

Currently, there is insufficient visibility of and access to high-quality, longitudinal, and well-characterized data and samples for AD and NDD research (5). Hundreds of cohorts across Europe, and more globally, hold significant amounts of data and samples that have been collected to answer specific questions related to AD and NDD research. But often, these datasets and samples are stored in different public institutions and/or in pharmaceutical companies, which tend to make them siloed, and/or not visible or accessible to external researchers. Another reason discovery can be difficult is the fragmentation of the technical tools, as they tend to cover a few aspects of discovery specific to the cohorts they are built for.

EPND aims to overcome these silos by building connections to existing platforms and leveraging and building on existing tools. Through MONTRA (6), for example, an application for data publishing and discovery, EPND can connect to existing catalogs like EMIF-AD, which includes 48 AD- and dementia-related cohorts representing over 85,000 patients (7). Through new and existing application programming interfaces (APIs), including Café Variome (8), EPND will enable data discovery. On the sample side, technologies, such as the MOLGENIS software (9) and the ELIXIR platform will support EPND's connection to sample catalogs and potentially biobanks via a federated approach. Facilitating discoverability of these resources by the larger research community can enable further analyses, as well as the surfacing of additional insights after the original study/trial leads have reported on initial findings.

EPND will also leverage an existing data platform developed by the Alzheimer's Disease Data Initiative (ADDI). The AD Workbench, one of EPND's critical pieces of infrastructure, will allow the platform to connect to an existing global network of data scientists and datasets. This connection will further promote collaboration and the generation of additional resources for a community of researchers from various neurodegenerative disciplines and research areas, and in doing so, advance and broaden their fields.

To meet the various technical and governance requirements of participating cohorts, EPND will offer a range of options by which cohorts can make their data and samples discoverable. A federated option can be offered to cohorts that must keep all patient-level data (including data about samples) local on premise or behind a firewall. A distributed configuration can enable cohorts to make their data temporarily available for analysis by permissioned users of the EPND platform, but the data and data about samples will be hosted within a secure local environment. A centralized option will also allow data to be temporarily available for analysis on the EPND platform, but the data (including data about samples) would be hosted in a secure public cloud environment. Methods to enable discovery, filtering, and querying of the various levels of data residing within these environments are currently being developed and refined to ensure privacy and security. Standard operating procedures for EPND use, as well as training, will clarify expectations for all users to ensure data and sample quality is kept high and regulatory frameworks are not violated. Researchers will be able to submit access requests for data and samples through the EPND platform. While options for the delivery of data to a researcher will depend on the technical and governance requirements of the cohorts, the EPND platform will provide secure, private, cloud-based workspaces where researchers can perform their analyses, save their work, and collaborate with others that have been granted permissioned access. In some cases, cohorts may be able to allow researchers to receive a copy of the data to be downloaded for local analysis. When the governance requirements will not allow researchers direct access to patient-level data, the EPND platform can support federated access to enable remote analyses via containerized scripts that are sent to the remotely hosted data and subsequently return the results.

Though existing technologies and infrastructures will be leveraged to establish the platform, any new tools and capabilities developed will be made open source, so the broader research community can benefit.

### Safeguarding legal and ethical sharing of sensitive data and samples

Keeping up to date with frequently changing regulations can be resource-intensive, and ensuring adherence to legal and ethical frameworks can be a hindrance to the exchange of valuable data and samples. Navigating these complex requirements for safe, legal, and ethical sharing can add a heavy burden to individual researchers/institutions. This dichotomy means that both researchers and legal requirements “have been challenged by the need to balance the twin aims of making data accessible to researchers while at the same time protecting the privacy of study participants and patients.” (10).

Assuming datasets and samples can be accessed, there will still be legal and ethical hurdles to be crossed, from understanding participant consent, to complying with data privacy requirements, to understanding changing country-specific requirements. In particular, researchers cite the GDPR as a particular obstacle to the secondary use of samples and/or data due to its lack of clarity on pseudonymization, controllership, derogations, and research exemptions.

EPND will develop a set of ethical, legal, and regulatory principles in the form of White Papers to guide platform design and the responsible discovery and sharing of data and samples. Over time, these principles will form the backbone of governance and data protection frameworks that facilitate research via EPND and reduce the burden of compliance for participating cohorts and users, all while ensuring the highest ethical standards are maintained across the platform. This guidance will aim to address the challenges and common principles associated with sharing human samples and associated data, while supporting compliance with GDPR by clearly identifying roles and responsibilities between cohort contributors, controllers, processors, and users of data. In addition, the project will consider the requirements regarding the quality of data and the principles related to sharing and access to data and samples when seeking biomarker qualification or drug approval from regulatory authorities. These White Papers, guidelines, and frameworks should facilitate and potentially streamline collaborations among users. Importantly, EPND will use its public-private expertise to co-create this guidance with input of patients and their caregivers, to build trust, awareness, and understanding within the community that represents the ultimate beneficiaries of AD and NDD research and innovation.

In addition to developing White Papers, guidance, and frameworks, EPND will also have dedicated resources to assist cohorts with understanding their ethical-legal readiness to share data and samples. Being able to help cohorts navigate these ethical and legal requirements is expected to further support contributions to the EPND platform.

### Enabling and driving broad-scale collaboration

Currently, few many-to-many or cross-disciplinary opportunities for cooperation across partners are leveraged, with the research ecosystem favoring simpler frameworks. This leads to siloing of datasets, with limited collaboration or reuse of data and samples. Consequently, the underutilization of these data and/or samples represents a significant missed opportunity for research.

EPND will promote and facilitate collaborations by connecting contributors of data and samples with users in the global community. By sharing their data and samples, scientists will advance their individual research while contributing to shared goals. Data and sample re-use could facilitate the development of more accurate disease progression models; identify novel risk factors and molecular drivers of disease; provide natural history studies for clinical trial arms; train artificial intelligence-based risk prediction algorithms; support the validation of biomarkers in diverse populations; and more (11). During proposal development, over 60 NDD cohorts across Europe acknowledged their willingness to collaborate with the EPND project. Being able to facilitate connections among these cohorts and others will be key to starting and maintaining the EPND community. Also, as noted above, the AD Workbench will offer access to a global network of interoperable datasets and will also enable collaboration with a broader community of users who may contribute ancillary or relevant data to the platform. Additionally, connecting to the AD Workbench will allow users access to secure cloud-based workspaces, with shared analytical tools, where they can work with other researchers and/or curate, harmonize, and analyze data. ADDI's AD Connect, a user-community resource that includes forum and knowledgebase features, is one potential option to promote and facilitate conversations and sharing of questions, knowledge, and lessons learned among EPND users and the broader global network of AD Workbench users.

Another key component to increasing collaboration is to increase the visibility and discoverability of cohorts, which otherwise would not have the opportunity to be part of a larger network. This increased visibility and discoverability will promote information and data and sample exchange among researchers. EPND could be best positioned to make a large number of cohort datasets discoverable and interoperable for future research, as heterogeneity in cohort datasets, linked to the use of different data models, sampling methods, and recruitment criteria, compounds these issues and further impedes the use of data and samples for large-scale research (12). Entry into EPND's catalog – in datasets or completed case studies – will require minimal effort on the part of contributors, making it easy for researchers to add value to the community, and ensuring they have opportunities to participate in studies before they are published.

### Ensuring sustainability after project completion

When projects and studies end, collected data and samples can become difficult to access by the scientific community. As such, public-private partnership projects are often challenged to consider what it would take to maintain and operate assets after initial funding periods have lapsed (13).

EPND will allow scientists to reuse data and samples for future projects, not only for original conclusions to be examined, verified, or occasionally corrected, but to facilitate the testing of new hypotheses. This extends the value of the original research investment (14), not only because it increases data validity, but because in promoting and facilitating reuse, greater value is extracted from original research, all while helping avoid unnecessary repetition of studies. As an example of extending the useful life of existing datasets, EPND plans to have mutually supported relationships with other IMI projects, such as EMIF, where the data will be made accessible and findable for the broader research community. Furthermore, as the EPND becomes a part of a global network with ADDI, there will be access to datasets from the EPAD project, including their Longitudinal Cohort Study (15). EPND will extend the useful life of the data collected by these projects, allowing researchers to access data and samples in one cohesive space, so they can build on each other in a useful fashion. The partnership with ADDI will also allow EPND to leverage the AD Workbench as an ongoing resource for the EPND user community without incurring additional expenses.

EPND will also establish an infrastructure that will be continuously refined through a series of case studies that utilize data and samples, including case studies on fluid biomarkers, clinical data, prospective longitudinal data, and digital biomarker data. These case studies will test the functionality and features of the platform, while simultaneously being able to adjust processes and platform elements to ensure the infrastructure is durable and can be self-sustaining. Learnings from case studies will include an understanding of operational and governance components required to select, permission, share, and process data and samples. Novel findings and new data generated from case studies will be made available on EPND, ensuring these resources can continue to be used for future research. EPND will also strive for regulatory validation of any potential biomarkers to enhance the utility of EPND and its overall goal to accelerate development of diagnostics and treatment of AD and other neurodegenerative diseases. Finally, there will be benchmarking exercises to compare EPND's offerings to other initiatives and platforms, and outreach to potential users to better understand how to attract and incentivize use of the platform.

## The ambition: Validate research that speeds up the fight against neurodegenerative diseases

The ultimate goal of EPND is to accelerate research into the discovery and validation of biomarkers to support development of diagnostics and disease-modifying therapies for Alzheimer's disease and other neurodegenerative diseases.

**Figure 1:**
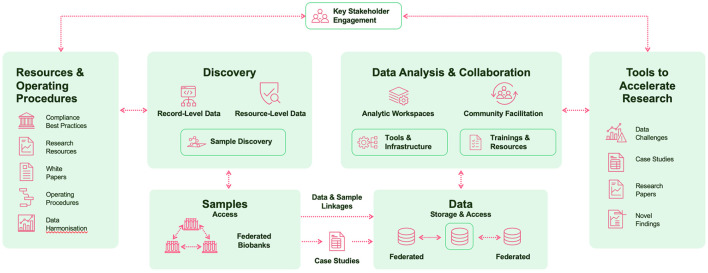
EPND programme overview.

The universal platform will enable the sharing, reuse and large-scale analysis of high-quality data and samples to accelerate biomarker discovery, development, and validation, while maintaining robust protection for the fundamental rights of data subjects. It will promote collaboration, harmonize a fragmented landscape, and ensure the highest legal and ethical standards are met, all while giving data and research additional usability, beyond original studies (Figure 1).

By creating a virtuous cycle of discovery, access, and re-use of data and samples to facilitate new research, as part of a sustainable process and infrastructure facilitating collaboration across a global community of users, EPND will be a scalable, sustainable solution to support more disruptive research on biomarkers and disease-modifying therapies for Alzheimer's disease and other neurodegenerative diseases in Europe, while opening a pathway to become a model scaled beyond Europe – and beyond AD and NDD themselves.

## Author contributions

NB, AB, PS, and PV jointly conceived and led the work, providing critical comments on manuscript drafts, and approving the final manuscript. All authors contributed to the article and approved the submitted version.

## Funding

This work was supported by funding from the Innovative Medicines Initiative 2 Joint Undertaking (JU) under grant agreement number 101034344 (EPND). The IMI JU receives support from the European Union's Horizon 2020 research and innovation programme and EFPIA.

## Conflict of interest

NB was employed by the company Gates Ventures. PS was employed by the company UCB Biopharma UK. The remaining authors declare that the research was conducted in the absence of any commercial or financial relationships that could be construed as a potential conflict of interest.

## Publisher's note

All claims expressed in this article are solely those of the authors and do not necessarily represent those of their affiliated organizations, or those of the publisher, the editors and the reviewers. Any product that may be evaluated in this article, or claim that may be made by its manufacturer, is not guaranteed or endorsed by the publisher.
